# Characterization of macrophages from schizophrenia patients

**DOI:** 10.1038/s41537-017-0042-4

**Published:** 2017-11-14

**Authors:** Paul R. Ormel, Hans C. van Mierlo, Manja Litjens, Miriam E. van Strien, Elly M. Hol, René S. Kahn, Lot D. de Witte

**Affiliations:** 1 Department of Translational Neuroscience, Brain Center Rudolf Magnus, University Medical Center Utrecht (BCRM-UMCU), P.O. Box 85500, Heidelberglaan 100, 3508 GA Utrecht, The Netherlands; 2 Department of Psychiatry, BCRM-UMCU, P.O. Box 85500, Heidelberglaan 100, 3508 GA Utrecht, The Netherlands; 3 Netherlands Institute for Neuroscience, An Institute of the Royal Netherlands Academy of Arts and Sciences, Meibergdreef 47, 1105 BA Amsterdam, The Netherlands

## Abstract

Genetic, epidemiological and *post mortem* studies have described an association between schizophrenia (SCZ) and the immune system. Microglia, the tissue-resident macrophages of the brain, not only play an essential role in inflammatory processes, but also in neurodevelopment and synapse refinement. It has therefore been hypothesized that aberrant functioning of these myeloid immune cells is involved in SCZ pathogenesis. Until now cellular research into the role of myeloid cells in SCZ has been limited to monocytes and functional assays are lacking. In this study we used monocyte-derived macrophages (mo-MΦs) as a model for macrophages and microglia in the CNS and examined two main functions: Inflammatory responses and expression and regulation of synapse refinement molecules. The expression of 24 genes involved in these key functions was assessed. Mo-MΦs were generated from 15 SCZ patients and 15 healthy controls. The cells were exposed to pro-inflammatory and anti-inflammatory stimuli (LPS, R848, IL-4 and dexamethasone), and the response was measured by qPCR and ELISA analyses. One of the genes of interest, *P2RX7* that is associated with psychiatric diseases, was significantly reduced in expression after LPS stimulation in SCZ patients. None of the other assessed characteristics were different in this functional screen between mo-MΦs from SCZ patients compared to controls. Although these data suggest that overall the function of macrophages in SCZ is not impaired, further studies with larger groups that enable the possibility to study clinical subgroups and perform additional screenings to asses the full phenotype of the mo-MΦs are needed to strengthen this conclusion.

## Pathogenesis: Limited signs of immune cell impairment in schizophrenia

Immune cells derived from schizophrenia patients exhibit similar functional characteristics to those from healthy individuals. Immunological function has repeatedly been linked to schizophrenia, yet exactly how the two interact is yet to be described. Aiming to clarify these molecular mechanisms, a team of researchers led by Paul Ormel, of the University Medical Center Utrecht, The Netherlands, took immune cells from patients and healthy individuals before assessing how the cells expressed their genetic material under normal conditions and after stimulation. For greater accuracy, the cells were changed into a cell type that resembles cells found in brain tissue. Ormel and his team found only one gene of interest that was expressed differently in cells from schizophrenia patients, which only differed during inflammatory stimulation. This is the first study investigating the function of these immune cells in schizophrenia.

## Introduction

Schizophrenia (SCZ) is a psychiatric disorder that is caused by the interplay between genetic and environmental factors. SCZ is generally viewed as a neurodevelopmental disorder that involves abnormal synapse development and functioning.^1^ The cause of these synaptic deficits is still largely unknown. One of the more recent hypotheses proposes that the synaptic deficits are partly caused by an aberrant function of the immune system.^2^

Immunological pathways have repeatedly been associated with SCZ. Single-nucleotide polymorphisms in immune genes are associated with the disease,^2,3^ the prevalence of immune-related disorders is higher in patients and their family members,^4^ and altered levels of immunological markers are measured in blood, cerebrospinal fluid and brain tissue of SCZ patients compared to controls.^5–7^ Which immunological pathways are affected in SCZ is still elusive as well as how these pathways contribute to synaptic deficits underlying SCZ pathogenesis.

Microglia and macrophages are the most prominent immune cells in the brain and belong to the myeloid type of immune cells, similar to monocytes. In the healthy brain, microglia reside in the brain parenchyma, whereas macrophages are found perivascular, in the choroid plexus and in the meninges.^8,9^ In many neurological diseases, blood-resident monocytes infiltrate the brain parenchyma and differentiate into monocyte-derived macrophages (mo-MΦs).^10^ All these myeloid cell subtypes express receptors to detect exogenous danger signals, such as pathogens, as well as endogenous inflammatory signals, such as cytokines, ATP and glucocorticoids.^11,12^ Triggering these receptors leads to an altered phenotype and the induction of a specific inflammatory response.^11,13^

Over the last decade it has become clear that microglia in the central nervous system (CNS) are not only involved in inflammatory processes but also play a role in synapse refinement by cell–cell interaction.^14^ The involved ligands and receptors that have been identified so far are all known for their role in the immune system, including components of the complement system (C1q; iC3b; C4b) and their receptors (CR1; CR3; CR4), fractalkine (CX3CL1) and its receptor CX3CR1, triggering receptor expressed on myeloid cells 2 (TREM2), ATP and its adrenergic receptors like P2×7, and CD47 and its receptor signal regulatory protein α (SIRPα/CD172α).^15^ Although synapse refinement has been attributed to microglia, it is likely that mo-MΦ, once in the brain, also interact with neurons since they also express TREM2, SIRPα, the complement receptors and chemokine receptors and synapse material has been found within mo-MΦs when co-cultured with neurons in vitro.^10,16^ The distinct roles of microglia and macrophages in synapse refinement remain poorly understood. It is important to note that some microglia receptors are low expressed on mo-MΦ, like CX3CR1 (ref. 17).

The density of cells expressing myeloid markers and the expression of some pro-inflammatory cytokines are increased in SCZ post mortem brain tissue, although results are heterogeneous.^5,7^ More indirect support for an altered function of myeloid cells in SCZ is derived from studies using peripheral blood monocytes where the expression of inflammatory genes and proteins was altered in cells from SCZ patients.^18,19^ Other studies provide a link between SCZ and molecules that are important for synapse refinement. This includes the association with copy number variations and increased expression in the brain of complement factor 4 (ref. 2), reductions in CX3CR1 expression in blood,^20^ and increased expression of TREM2 in leukocytes.^19^

Together these findings have led to the hypothesis that impaired functioning of myeloid immune cells in the brain may be involved in the pathogenesis of SCZ. Genetic variants and/or the interaction with environmental factors may shift myeloid cells towards an altered phenotype, thereby erroneously switching on or off processes that underlie the disease, such as inflammatory processes and involvement in synapse regulation. Functional studies with microglia or macrophages from SCZ patients and controls that could support this hypothesis are lacking so far. Since it is not possible to obtain primary microglia and macrophages from the CNS from living individuals, we performed a functional screen of monocytes from SCZ patients and controls that were differentiated into macrophages in vitro. This model, inducing the so-called monocyte-derived macrophages (mo-MΦs), is widely used in immunological research for functional experiments on macrophages.^21^ We screened the mo-MΦs for (1) The expression of genes involved in inflammation and the response to pro-inflammatory and anti-inflammatory molecules and (2) The expression and regulation of synapse refinement-related receptors.

## Results

### Mo-MΦs phenotype

Monocytes were isolated from 15 patients and 15 controls and differentiated into mo-MΦs in vitro. The demographics of the subjects are depicted in Table 1. All cultures showed the characteristic morphology of mo-MΦs (data not shown) and expressed the expected markers for macrophages, including *MRC1* and *CD200R1* (Table 2). Table 2 shows the expression levels of 24 genes that are involved in inflammation and synapse refinement in mo-MΦs compared to controls. None of the genes were differently expressed in SCZ patients compared to controls when analyzed with Mann–Whitney *U* tests. No confounding effect of gender was detected.

**Table 1:** The subject demographics including: sex, age, ethnicity, cannabis usage, DSM-IV-based diagnosis, and the antipsychotic medications used

Subjects (*n*)	Male/ female	Mean age (st. dev)	Ethnicity (*n*)	Cannabis basal level (*n*)	Cannabis at moment of blood withdrawal (*n*)	DSM-IV (*n*)	Antipsychotics at moment of blood withdrawal (*n*)
Controls (15)	8/7	30 (11)	Caucasian (14) Mixed (1)	No (15)	No (15)	–	–
Patients (15)	13/2	31 (6)	Caucasian (13) Mixed (1) Turkish (1)	No (9) Yes (6)	No (14) Yes (1)	295.xx(15)	Atypical Clozapine(5) Quetiapine(1) Olanzapine(2) Risperidone (1) Other (5) None (1)

*st. dev* standard deviation

**Table 2:** The median, interquartile range (25–75%), Mann–Whitney *U*-value and *p*-value of the mRNA expression values of 24 genes important for monocyte-derived macrophage functioning^40^ determined by qPCR analyses compared between patients (*n* = 15) and controls (*n* = 15)

	Gene (protein)	Function	Control median (25–75%)	Patient median (25–75%)	Mann-Whitney *U*-value	*p*-value
Inflammatory response proteins	*CD45* (CD45)	Activation marker	23 (18–29)	22 (16–30)	106.5	0.81
	*HLA-DRA* (HLA-DRA)	Activation marker	438 (289–684)	480 (353–676)	99	0.59
	*CD200R1* (CD200R)	Inhibits activation^41,42^	1 (0.05–389)	1 (0.2––359)	101.5	0.66
	*MRC1* (CD206)	Mannose receptor	125 (76–149)	132 (102––189)	94	0.46
	*CD209* (DC–SIGN)	C–type lectin	1.2 (0.3–1.8)	0.8 (0.6–1.2)	101	0.64
	*CD163* (CD163)	Scavenger receptor	70 (36–149)	71 (44–134)	110.5	0.94
	*FCGR3A* (CD16)	Immunoglobulin receptor	86 (44–99)	58 (45–85)	90	0.36
	*TLR2* (CD282)	Toll-like-receptor that binds gram-positive bacteria	10 (8–13)	10 (6–15)	103.5	0.72
	*TLR3* (CD283)	Toll-like receptor that binds dsRNA	0.8 (0.5–12.6)	1 (0.3–15)	105.5	0.78
	*TLR4* (CD284)	Toll-like receptor that detects LPS on gram-negative bacteria	27 (0.5–32.1)	21 (0.6–41)	101.5	0.66
	*CD14* (CD14)	Co-receptor of TLR4	200 (90–1050)	188 (96–1203)	112	>0.99
	*TNF* (TNFα)	Pro-inflammatory cytokine	6 (4–8)	4 (3–4)	68.5	0.07
	*IL1B* (IL-1β)	Pro-inflammatory cytokine	4 (2–6)	2 (2–3)	78	0.16
	*IL6* (IL-6)	Pro-inflammatory cytokine	0.14 (0.02–0.24)	0.06 (0.01–0.45)	107	0.82
	*IFNB1* (IFNβ)	Activates innate immune cells, important for viral infection resistance	0.01 (0.00–0.05)	0.01 (0.00–0.08)	91.5	0.32
	*TGFB1* (TGFβ)	Anti-inflammatory cytokine	238 (207–256)	257 (201–290)	89	0.34
	*IL10* (IL-10)	Anti-inflammatory cytokine	4 (2–7)	3 (2–5)	100.5	0.63
	*CCL2* (CCL2)	Chemokine that attracts immune cells	196 (106–307)	172 (90–262)	100	0.62
Synapse refinement proteins	*P2RX7* (P2X7)	Adrenergic receptor, specifically receptive for ATP, a danger signal that can be secreted from neurons and affects microglia functioning^43^	23 (13–29)	21 (17–27)	112	0.99
	*TREM2* (TREM2)	Regulator of pro-inflammatory genes, induces phagocytosis via DAP12 (ref. 41)	209 (138–346)	184 (167–323)	104	0.74
	*CX3CR1* (CX3CR1)	Fractalkine receptor, involved in synaptic refinement^44^	0.01 (0.00–0.04)	0.02 (0.00–0.14)	76.5	0.08
	*ITGAM* (CD11b)	Subunit of complement receptor 3, which is important for synaptic refinement^45^	58 (37–80)	54 (30–68)	102	0.68
	*TYROBP* (DAP12)	Important for synaptic refinement and ROS production for pathogen destruction, signaling partner of CR3 & TREM2^41^	1469 (1228–1939)	1765 (1462–2501)	82	0.22
	*SIRPA* (CD172-α)	CD47 receptor that inhibits synaptic refinement^46^	400 (285–486)	334 (209–497)	94	0.46

### Pro-inflammatory and anti-inflammatory responses

**Fig. 1:**
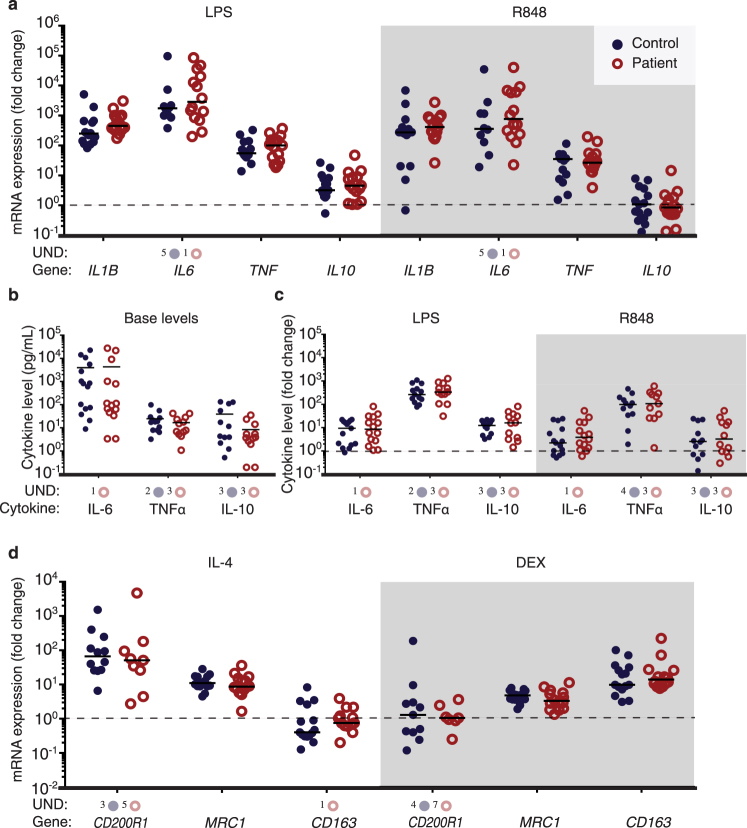
mRNA expression levels of inflammatory response genes and cytokines secreted after inducing a pro-inflammatory or anti-inflammatory phenotype in mo-MΦs of SCZ patients () and controls (). mRNA levels of inflammatory response genes after 6-h stimulation with LPS or R848 were not different between patients (*n* = 15) and controls (*n* = 15) **a** Five or one samples gave undetermined values for *IL6* expression in the control and patient group, respectively. The basal cytokine levels (**b**) and fold change values after pro-inflammatory stimulation (**c** LPS and R848) of IL-6, TNFα and IL-10 in the medium were similar between SCZ patients and controls. The base level of IL-6 was undetermined for one patient sample. At base level and after LPS stimulation two and three undetermined values were present for TNFα secretion in controls and patients samples, respectively. After R848 stimulation four and three samples gave undetermined values for controls and patients, respectively. Both in the patient and the healthy control group, three samples gave undetermined values for IL-10 secretion. There was no difference measured between the patient (*n* = 14) and control group (*n* = 15) on mRNA levels of inflammatory response genes after 72-h anti-inflammatory stimulation with IL-4 or dexamethasone (**d**). After IL-4 stimulation three samples gave undetected values in *CD200R1* expression in the control group and five in the patient group. *CD200R1* mRNA values of four control and seven patient samples were undetermined after dexamethasone stimulation. One patient sample was undetected for *CD163* expression after IL-4 stimulation. mRNA expression was analyzed with qPCR and cytokine secretion with ELISA. The mRNA values **a**, **d** were first normalized (2^Δ*ct*^) with *GAPDH* as reference gene after which they were divided by the value of the sample without stimulation (dotted line) resulting in the fold change. The median is depicted with a black bar. Mann–Whitney *U* tests were used to compare the SCZ patients with the healthy controls. UND undetermined value

Lipopolysaccharide (LPS) and R848 induced increased expression levels of *IL1B*, *IL6*, *TNF* and *IL10* mRNA (controls *n* = 15, SCZ patients *n* = 15) (Fig. 1a) and secretion of IL-6, TNF-α and IL-10 proteins (Fig. 1b, c), as expected.^13,17,22^ IL-1β protein secretion was below detection levels before and after stimulation (data not shown).

From one patient there were not enough cells available to perform the 72-h stimulation experiments (controls *n* = 15, SCZ patients *n* = 14). IL-4 or dexamethasone induced the expected^17^ anti-inflammatory response, as IL-4 resulted in increased mRNA expression of *CD200R1* and *MRC1*, whereas dexamethasone increased the expression of *CD163* and *MRC1* (Fig. 1d). The response to pro-inflammatory and anti-inflammatory stimulation was not different between patients and controls when analyzed with Mann–Whitney *U* tests (Fig. 1a–d). No confounding effect of gender was detected.

### Receptors involved in synapse refinement

**Fig. 2:**
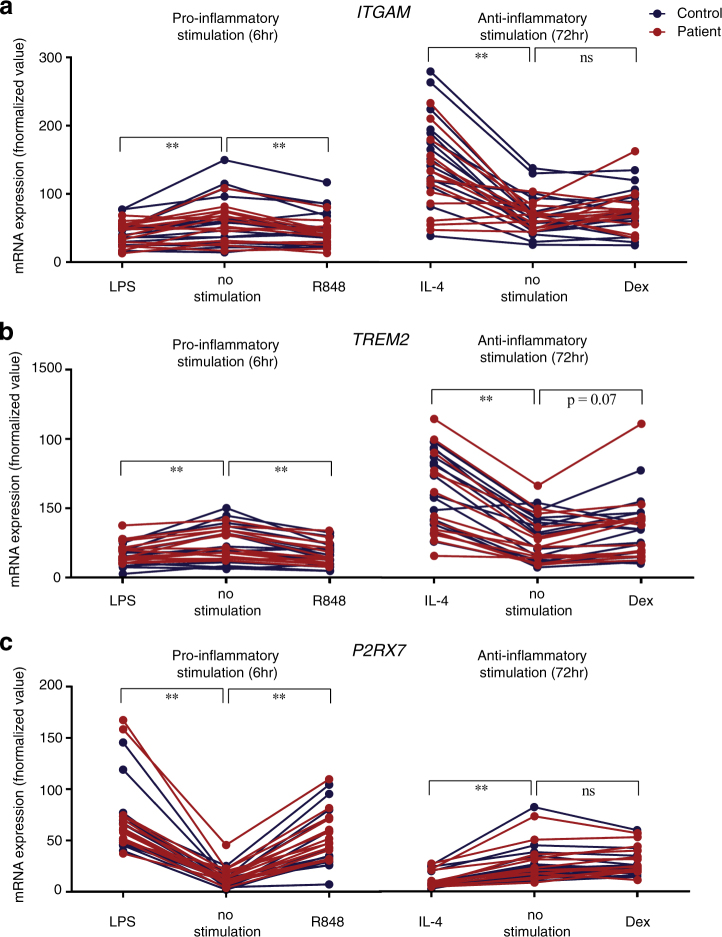
mRNA expression levels of synapse refinement receptors *ITGAM* (**a**), *TREM2* (**b**) and *P2RX7* (**c**) in mo-MΦs was compared between a non-stimulated state and after pro-inflammatory (*n* = 30) or anti-inflammatory (*n* = 29) phenotype induction. The pro-inflammatory phenotype was induced by 6-h stimulation with LPS or R848 and the anti-inflammatory phenotype was induced by 72-h stimulation with IL-4 or dexamethasone. *ITGAM* (**a**) and *TREM2* (**b**) mRNA levels were significantly decreased after LPS (*p* < 0.01) and R848 (*p* < 0.01), increased after IL-4 (*p* < 0.01) and not altered after dexamethasone stimulation. *P2RX7* (**c**) mRNA levels were not altered after dexamethasone stimulation, but significantly decreased after IL-4 (*p* < 0.01) and increased after LPS (*p* < 0.01) and R848 stimulation (*p* < 0.01). We did not distinguish between the schizophrenia patients () and healthy controls () in the statistical analyses. One patient sample gave an undetermined value for *P2RX7* expression after dexamethasone stimulation. mRNA expression was analyzed with qPCR. All values were normalized (2^Δ*ct*^) with *GAPDH* as the reference gene after which the value was multiplied by 1000 to ease visualization. Friedman’s ANOVA tests were used to compare the pro-inflammatory or anti-inflammatory responses to the non-stimulated state. (*p < 0.05; **p < 0.01)

The expression levels of *ITGAM* and *TREM2* were significantly up regulated after IL-4 stimulation (*ITGAM*: Dunn’s test −37 *p* < 0.0001, *TREM2*: Dunn’s test −50 *p* < 0.0001), but not dexamethasone (*ITGAM*: Dunn’s test −5 with *p* > 0.999; *TREM2*: Dunn’s test −16 *p* > 0.999) and down regulated after LPS or R848 stimulation in the whole group (Fig. 2a, b) (*ITGAM*: Dunn’s test for LPS: 29 with *p* < 0.001 and for R848: 22 with *p* < 0.01; *TREM2*: Dunn’s test for LPS: 27 with *p* < 0.01, and for R848: 39 with *p* < 0.0001). In contrast, *P2RX7* was significantly up regulated after LPS or R848 stimulation (*P2RX7*: Dunn’s test for LPS: −52 with *p* < 0.0001, and for R848: −32 with *p* < 0.0001) (Fig. 2c). *P2RX7* was significantly down regulated after IL-4, but not dexamethasone, stimulation in the whole group (*P2RX7*: Dunn’s test for IL-4: 38 with *p* < 0.0001, for dexamethasone: −8 with *p* = 0.57) (Fig. 2c).

**Fig. 3:**
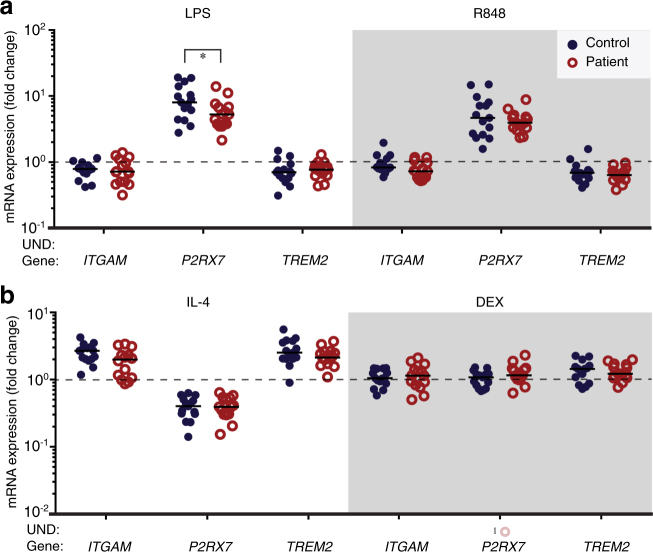
Pro-inflammatory or anti-inflammatory induced changes in mRNA expression levels of proteins important for synapse refinement compared between SCZ patients () and controls (). There was no difference in expression levels of the genes between patients (*n* = 15) and controls (*n* = 15) after the pro-inflammatory phenotype was induced by 6-h stimulation with LPS or R848 (**a**), except for *P2RX7* that was significant lower expressed after LPS stimulation in patients versus controls (*U* = 65, *p* = 0.049). The anti-inflammatory phenotype was induced by 72-h stimulation with IL-4 or dexamethasone and was also not different between patients (*n* = 14) and controls (*n* = 15) (**b**). One patient sample gave an undetermined value for *P2RX7* expression after dexamethasone stimulation. mRNA expression was analyzed with qPCR. All values were first normalized (2^Δ*ct*^) with *GAPDH* as the reference gene after which they were divided by the value of the sample without stimulation (dotted line) resulting in the fold change. Mann–Whitney *U* tests were used to compare the SCZ patients with the healthy controls. UND undetermined value (*p < 0.05)

When comparing the expression of genes involved in synapse refinement after pro-inflammatory and anti-inflammatory stimulation between SCZ patients and controls, only *P2RX7* was significantly altered in mo-MΦs after stimulation with LPS (Fig. 3a, b) (*U* = 65, *p* = 0.049), but not after R848 stimulation (*U* = 97, *p* = 0.539) or the anti-inflammatory stimulations (IL4: *U* = 103, *p* = 0.949; dexamethasone: *U* = 72, *p* = 0.254).

## Discussion

Impaired functioning of myeloid immune cells has been hypothesized to be involved in SCZ pathogenesis. In this study we therefore investigated the phenotype and function of mo-MΦs in SCZ. We did not detect significant alterations in expression of a panel of genes involved in inflammatory responses and synapse refinement. Except for an altered expression level of purinergic receptor P2X7 after pro-inflammatory stimulation with LPS, no irregularities were detected in the functional response to pro-inflammatory and anti-inflammatory phenotype induction between patients and controls.

The P2X7 receptor is important for purinergic intercellular signaling inside and outside the CNS. Although several genes in close proximity to *P2RX7* in chromosomal region 12q24.21-33 are associated with SCZ and it is linked to other psychiatric diseases,^23,24^ our study shows an effect of *P2RX7* in SCZ. Interestingly, this effect only became visible upon a functional challenge with LPS. It suggests that the response of myeloid cells to inflammatory stimuli might be affected in SCZ patients, but further research is needed especially since inflammatory stimulation with R848 did not show this effect. For some of the genes (*TREM2, CX3CR1, HLA-DRA, IL1B, IL6*, and *TNF*) that were in our panel, altered expression levels have been described in leukocytes and *post mortem* brain tissue of SCZ patients.^5,7,19,25^ Importantly, these other studies analyzed different cell types or full brain tissue, while we used mo-MΦs. This may well explain the seeming inconsistency in results. Mo-MΦs more closely resemble the phenotype of myeloid cell subsets (macrophages and microglia) in the brain than monocytes or other leukocyte subsets.^8^ Another advantage of using mo-MΦs is that a homogeneous cell population is obtained. For peripheral blood mononuclear cells (PBMCs) or total brain tissue differences the cell type composition always need to be taken into account. In addition, the 7-day differentiation protocol needed for the generation of mo-MΦs presumably also diminishes the effect of disease-related confounders, such as medication,^26^ psychosocial stress^27^ and sleep disturbances.^28,29^ On the other hand, in vitro culturing cells might also diminish a disease-related phenotype.

In this study we investigated the phenotype and function of mo-MΦs in SCZ. The cell model that we applied here has been used in many immunological studies and has retrieved important information on the role of tissue-resident macrophages in health and disease.^21^ It is important to also realize the limitations of this cell model. First of all, similar to iPSC-derived cells, an in vitro cell model like this will never fully match the primary cells in the brain as the environmental stimuli the cell received will not be completely similar; second, the main population of resident myeloid cells in the non-inflamed brain consists of microglia, whereas our model represents the blood-borne macrophages. Although we expect that inflammatory responses and the interaction with neurons are similar between microglia and macrophages, they show important differences in phenotype.^17,30^ Therefore it is yet unclear how the results from the present study can be translated and follow-up studies are needed to replicate these findings in microglia from patients with SCZ.

Another point for discussion is the sample size. This was based on the power calculation and other studies assessing functional abnormalities in SCZ and other diseases.^31,32^ Although we hypothesized that dysfunctional myeloid cells in SCZ could be a collective hallmark in SCZ, the disease is often viewed as a disease with a heterogeneous pathology.^33^ In addition, the use of antipsychotics might have influenced the characteristics of the examined macrophages. To further examine this, we performed additional analyses only including patients that were using atypical antipsychotics (*n* = 9). This subgroup also did not reveal any alteration in the functioning of mo-MΦs in SCZ (data not shown). As there is still variation between the antipsychotics from this group, future similar studies should strive to include patients that are medication-free or using the same type of antipsychotic medication.

In summary, in this study we report a functional screening of myeloid cells of SCZ patients. We decided to tackle the attribution of myeloid immune cells in SCZ by focusing on inflammatory responses and the regulation of synapse refinement molecules, and we did not find indications for functional impairments in mo-MΦs of SCZ patients. It is possible that other cellular mechanisms in myeloid cells are involved in SCZ pathogenesis, for which in future studies an explorative non-hypothesis-driven RNAseq approach could be helpful. A bystander effect could be an explanation for the alterations found in myeloid cells in SCZ in previous studies. Moreover, it cannot be excluded that involvement of myeloid cells in SCZ is confined to microglia, or that our sample size was not large enough to detect subtle differences or alterations in subgroups of macrophages. Further studies are therefore needed to extend this initial functional screen.

## Methods

### Clinical samples

Blood samples were derived from 15 SCZ patients and 15 healthy controls as part of the multicenter longitudinal Genetic Risk And Outcome of Psychosis (GROUP) study, which started in The Netherlands in 2006 (ref. 34). The sample size was based on the power calculation (explained below) and other functional studies using mo-MΦs in other diseases or other cell types in SCZ.^31,32^ Inclusion criteria for patients were: fluent in Dutch and diagnosis of SCZ (DSM-IV classification 295.xx) at follow-up according to the Comprehensive Assessment of Symptoms and History or Schedules for Clinical Assessment in Neuropsychiatry interview. Eligible healthy controls had to meet the following criteria: fluent in Dutch, no history of a lifetime psychotic disorder or lithium use, and no first-degree or second-degree family member with a lifetime psychotic disorder. The human ethics committee of the University Medical Center Utrecht approved this study. All the subjects included in the study provided written informed consent before participating. Blood samples for this study were collected during a follow-up visit in 2013. Potential confounding factors like cannabis abuse were noted based on clinical sample interrogation and/or urine sample testing and visualized together with the other demographics of the subjects in Table 1.

### Generation of mo-MΦs

Blood was collected in heparinized tubes after which PBMCs were isolated by performing a Ficoll-Paque™ PLUS gradient (GE Healthcare, UK) within 4 h after the blood draw. PBMCs were harvested and the monocyte population was enriched via positive selection with CD14-coupled magnetic micro beads according to the manufacturers protocol (Miltenyi Biotec GmbH, Germany). The CD14^+^ cells were suspended in culture medium (Gibco® RPMI 1640 substituted with 100 Units/mL penicilin, 100 μg/mL streptomycin and 2 mM L-glutamin (BioWhittaker, Belgium)), collected in cryovials, slowly frozen in freezing medium (50% culture medium, 40% fetal bovine serum and 10% dimethylsulfoxide) and after one night at −80 °C, stored in liquid nitrogen until further use. To generate mo-MΦs, monocytes were thawed, immediately washed and resuspended in culture medium supplemented with 10% human serum (Sanquin, The Netherlands), and plated (1.25*10^^5^ cells/well) in 48-well Corning® Costar® cell culture plates (Corning, NY) for seven days to generate mo-MΦs.^35^ The cells were then used for qPCR analysis or pro/anti-inflammatory stimulation assays.

### Pro/anti-inflammatory phenotype induction

Inflammatory responses were investigated as previously described.^17^ To investigate pro-inflammatory immune responses, mo-MΦs were stimulated for 6-h with 100 ng/mL lipopolysaccharide (LPS) derived from Escherichia coli (0111:B4, Sigma-Aldrich, MO) or 1 μg/mL resiquimod (R848) (InvivoGen, CA), an agonist for TLR7/8 at day 7. Anti-inflammatory responses were investigated by stimulating the cells after differentiation for 72-h with 40 ng/mL interleukin-4 (IL-4) (Miltenyi Biotec GmbH, Germany) or 1 μM dexamethasone (Sigma Aldrich, MO) at day 7. After stimulation, the cells were lysed and stored for qPCR analysis and the medium was harvested to measure the secretion of cytokines.

### RNA isolation and qPCR analysis

RNA was isolated with the RNeasy mini kit (Qiagen, Germany). Cells were lysed using 350 μL RLT buffer and 1% β-mercaptoethanol at room temperature (Merck-Schuchardt, Germany) and stored at −80 °C until the RNA was isolated as prescribed in the protocol provided by the manufacturer. The purified RNA was diluted in 32 μL RNase-free water and RNA concentration and purity determined with the NanoDrop 2000 (Thermo scientific, MA). Afterwards the samples were stored at −80 °C until the cDNA synthesis was performed using the Quantitect reverse transcriptase kit (cat no: 205311, Qiagen, Germany) as described in the manufacturers protocol. The amount of cDNA used per qPCR reaction was based on an input of 3.5 ng total RNA in a final volume of 10 µL. Master mixes were prepared and per reaction it consisted of 5 µL SYBRgreen PCR Master Mix (Roche; Life Technologies Corporation, Grand Island, NY), and 1 µL primer mix (2 pmol/mL) was added. The qPCR reaction (95 °C for 10 min and then 40 cycles of 15 s 95 °C and 1 min 60 °C) was performed on the QuantStudio™ 6 Flex Real-Time PCR System (Life Technologies Corporation, NY). All intron-spanning primers were designed online in the primer design tools of NCBI or Primer Express (Table 3). Values were normalized (2^Δ*ct*^) to GAPDH as the reference gene.

**Table 3:** Forward and reverse primer sequences that were used for the quantitative polymerase chain reactions (qPCR) analyses

Gene	5′-Forward primer-3′	5′-Reverse primer-3′
*GAPDH*	TGCACCACCAACTGCTTAGC	GGCATGGACTGTGGTCATGA
*CD45*	GCAGCTAGCAAGTGGTTTGTTC	AAACAGCATGCGTCCTTTCTC
*HLA-DRA*	CCCAGGGAAGACCACCTTT	CACCCTGCAGTCGTAAACGT
*CD200R1*	GTGGCAGGTCACGGTAGACA	GAGCAATGGCACAGTGACTGTT
*MRC1*	TGCAGAAGCAAACCAAACCTGTAA	CAGGCCTTAAGCCAACGAAACT
*CD209*	GATTCCGACAGACTCGAGGA	CCTGACTTATGGAGCTGGGG
*CD163*	TTTGTCAACTTGAGTCCCTTCAC	TCCCGCTACACTTGTTTTCAC
*FCGR3A*	GACAGCGGCTCCTACTTCT	ATGGTTGACACTGCCAAACCT
*TLR2*	ATCCTCCAATCAGGCTTCTCT	GGACAGGTCAAGGCTTTTTACA
*TLR3*	CAAACACAAGCATTCGGAATCTG	AAGGAATCGTTACCAACCACATT
*TLR4*	AGTTGATCTACCAAGCCTTGAGT	GCTGGTTGTCCCAAAATCACTTT
*CD14*	ACAGGGCGTTCTTGGCTCGC	CGGGAAGGCGCGAACCTGTT
*TNF*	TGGAGAAGGGTGACCGACTC	TCACAGGGCAATGATCCCAA
*IL1B*	TTTGAGTCTGCCCAGTTCCC	TCAGTTATATCCTGGCCGCC
*IL6*	TGCAATAACCACCCCTGACC	TGCGCAGAATGAGATGAGTTG
*IFNB1*	AAACTCATGAGCAGTCTGCA	AGGAGATCTTCAGTTTCGGAGG
*TGFB1*	CAATTCCTGGCGATACCTCAG	GCACAACTCCGGTGACATCAA
*IL10*	TCAAGGCGCATGTGAACTCC	GATGTCAAACTCACTCATGGCT
*CCL2*	CAGCCAGATGCAATCAATGCC	TGGAATCCTGAACCCACTTCT
*P2RX7*	TCTTCCGAGAAACAGGCGAT	CCAACGGTCTAGGTTGCAGT
*TREM2*	TCAGGAAGGTCCTGGTGGA	GGGTGGGAAGGGGATTTCTC
*CX3CR1*	CGCGCAATCATCTTGGAGAC	CATCGCGTCCTTGACCCAT
*ITGAM*	TGCTTCCTGTTTGGATCCAACCTA	AGAAGGCAATGTCACTATCCTCTTGA
*TYROBP*	CGGAAACAGCGTATCACTGAG	TACGGCCTCTGTGTGTTGAG
*SIRPA*	GGCCTCAACCGTTACAGAGAA	GTTCCGTTCATTAGATCCAGTGT

### Primer panels for mo-MΦ characterization

We analyzed the mRNA expression of a panel of 24 genes to analyze phenotypic differences between mo-MΦ from SCZ patients versus controls. We selected the genes by studying recent review papers.^11,12,15^ This panel included genes that are involved in inflammatory responses (*CD45, HLA-DRA, CD200R1, MRC1, CD209, CD163, FCGR3A, TLR2, TLR3, TLR4, CD14, TNF, IL1B, IL6, IFNB1, TGFB1, IL10*, and *CCL2*) and synapse refinement (*P2RX7, TREM2, CX3CR1, ITGAM, TYROBP*, and *SIRPA*) (Table 2).

To asses and compare the pro-inflammatory response between mo-MΦ from SCZ patients versus controls, the gene expression of *IL1B*, *IL6*, *TNF* and *IL10* was determined, as these genes are expected to be up regulated after a pro-inflammatory stimulus.^13,17,22^ To assess the anti-inflammatory response, the expected up regulation^17^ of genes *CD200R1*, *MRC1*, and *CD163* was analyzed.

In addition we compared the regulation of expression of genes involved in synapse refinement after pro-inflammatory or anti-inflammatory stimuli between SCZ patients and healthy controls. We selected the genes (*ITGAM, TREM2*, and *P2RX7)* that have been described before to be responsive to or pro-inflammatory or anti-inflammatory stimulation.^36–39^ We first tried to replicate the earlier described effects on the whole group after which we looked for differences between SCZ patients and controls.

### Cytokine analysis

The concentrations of IL-1β, IL-6, TNFα and IL-10 in the medium of the mo-MΦs that were stimulated with LPS and R848 were analyzed using the anti-human IL-1β, IL-6, TNFα and IL-10 enzyme-linked immune-assay (ELISA) Ready-Set-Go® kits as prescribed by the manufacturer (eBioscience, CA). The samples were analyzed with an optical density (OD) of 450 nm with the Varioskan™ Flash (Thermo Fisher Scientific, MA). The blank value was subtracted from all the sample values and the standard curve was determined by using the values of the standard samples.

### Statistics

To determine the sample size a power calculation was performed on data of mRNA expression in mo-MΦs in a pilot study. G*Power software version 3.1 (G*Power Version 3.1.9.2, GE) was used with an α of 0.05 and a power of 0.8. This resulted in the requirement of at least eight subjects per group. Other statistical analyses were performed with GraphPad Prism software version 5 (GraphPad Software, CA) and R version 3.0.2. (CRAN: https://www.r-project.org/). The datasets were tested for a potential confounding effect of gender. All analyses were also performed with a subgroup of patients that were using atypical antipsychotics to exclude that we mask disease-related differences by including patients that use different types of antipsychotics. Because the data were not normally distributed we used Mann–Whitney *U* tests to analyze the patient versus control differences (expression levels) and Friedman’s ANOVA tests with Dunn’s multiple comparison post hoc test to analyze the stimulation assays as indicated in the results section and figure legends. A significance level of *p* < 0.05 was used.

### Data availability

Data available on request from the authors.
